# The response prediction and prognostic values of systemic inflammation response index in patients with advanced lung adenocarcinoma

**DOI:** 10.1111/1759-7714.14893

**Published:** 2023-05-02

**Authors:** Ran Zuo, Fuyi Zhu, Cuicui Zhang, Jincheng Ma, Jinliang Chen, Ping Yue, Jinfang Cui, Yu Wang, Peng Chen

**Affiliations:** ^1^ Department of Thoracic Oncology Lung Cancer Diagnosis and Treatment Center, Tianjin Medical University Cancer Institute and Hospital Tianjin China; ^2^ National Clinical Research Center for Cancer, Key Laboratory of Cancer Prevention and Therapy Tianjin's Clinical Research Center for Cancer Tianjin China; ^3^ Department of Pediatric Oncology Tianjin Key Laboratory of Cancer Prevention and Therapy, Tianjin Medical University Cancer Institute and Hospital, National Clinical Research Center for Cancer, Tianjin's Clinical Research Center for Cancer Tianjin China

**Keywords:** advanced lung adenocarcinoma, biomarkers, predictive, prognostic, systemic inflammation response index

## Abstract

**Purpose:**

This study aimed to assess the response prediction and prognostic values of different peripheral blood cell biomarkers for advanced lung adenocarcinoma (LUAD) patients receiving first‐line therapy.

**Methods:**

Patients diagnosed with advanced LUAD as well as healthy controls and patients with benign pulmonary diseases were collected in this retrospective study. Propensity score matching (PSM) was performed in a 1:1 ratio. Survival state was estimated by the Kaplan–Meier method and the Cox proportional hazard model was used to assess the prognostic factors.

**Results:**

Compared with the control groups, the level of peripheral blood leucocyte, neutrophil, monocyte, platelet, and neutrophil to lymphocyte ratio, monocyte to lymphocyte ratio, platelet to lymphocyte ratio, and systemic inflammation response index (SIRI) were higher in LUAD patients (all *p* < 0.001). Some inflammatory markers decreased at the time of optimal response and then increased again as the disease progressed. Multivariate analysis revealed that SIRI and lactate dehydrogenase (LDH) were independent prognostic factors no matter before or after PSM analysis. Area under the curve (AUC) of SIRI and LDH were 0.625 (*p <* 0.001) and 0.596 (*p* = 0.008), respectively. When SIRI and LDH were combined, the AUC reached 0.649 (*p* < 0.001).

**Conclusions:**

Pretreatment SIRI was an independent prognostic factor of progression free survival (PFS) in advanced LUAD patients. Dynamic monitoring of inflammatory index changes could help to predict therapeutic efficacy. The combination of SIRI and LDH is expected to be a promising clinically accessible biomarker in the future.

## Graphical abstract

The systemic inflammation response index (SIRI), which integrates different inflammatory cells, has certain diagnostic and prognostic value for lung adenocarcinoma patients. The dynamic changes of SIRI during treatment could help reflect therapeutic efficacy. SIRI combined with lactate dehydrogenase (LDH) improved the diagnostic efficiency and might be a better predictive marker for lung adenocarcinoma patients.

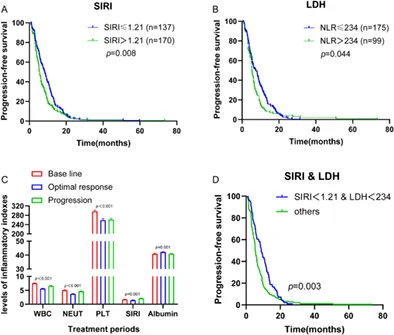

## INTRODUCTION

Lung cancer is one of the most common malignancies worldwide, with an overall high mortality rate, and non‐small‐cell lung cancer (NSCLC) accounts for 85% of lung cancers.^1^, ^2^ According to pathological type, NSCLC is divided into three major subtypes, of which lung adenocarcinoma (LUAD) is the most common subtype, accounting for about 50%, followed by lung squamous cell carcinoma and large cell lung cancer, 40% and 10%, respectively.^3^ The majority of LUAD cases present as metastatic disease on diagnosis, and the prognosis among these patients is grim.^4^ With the advent of precision treatment of LUAD, targeted therapy targeting driver genes have become an important part of advanced LUAD treatment and have significantly improved the prognosis of advanced LUAD.^5^ Although systemic treatment, including chemotherapy, molecular targeted therapy, and immunotherapy, has been extensively established, the outcomes of LUAD patients remain poor. There is therefore an urgent need for biomarkers which can predict treatment outcomes and so help to identify the patients most likely to benefit from treatment.

Inflammatory reaction and immune surveillance are important features of the tumor microenvironment that are involved in the development and therapeutic effect of cancer.^6^ Hematological inflammatory parameters such as neutrophils, lymphocytes, monocytes, and platelets can reflect immune status and have been reported to be effective predictors of prognosis in different tumor models. New inflammation indexes based on the integration of these conventional inflammatory parameters, such as the neutrophil to lymphocyte ratio (NLR), the monocyte to lymphocyte ratio (MLR), the platelet to lymphocyte ratio (PLR), the systemic immune‐inflammation index (SII), the prognostic nutritional index (PNI), the modified Glasgow prognostic score (mGPS), and the controlling nutritional status (CONUT) score, which represent nutritional and inflammatory status, have shown potential prognostic value in several studies.^7^, ^8^, ^9^, ^10^, ^11^

It has recently been reported that the novel systemic inflammation response index (SIRI), which integrates different inflammatory cells (neutrophils, monocytes, and lymphocytes), has been proved to be a promising prognostic predictor in different cancers, including gastric cancer, glioblastoma, breast cancer, esophageal squamous cell carcinoma, pancreatic cancer, nasopharyngeal carcinoma, and cervical cancer.^12^, ^13^, ^14^, ^15^, ^16^, ^17^, ^18^ This integrated indicator may comprehensively reflect the balance of host immune and inflammatory status compared with NLR, MLR, and PLR. Another biomarker used in the follow‐up of cancer treatment is lactate dehydrogenase (LDH), an enzyme that plays an essential role in anaerobic glycolysis and induces cell proliferation. A systematic review reported that elevated LDH was correlated with poor clinical outcome and resistance to therapy in a variety of tumors.^19^

However, the prognostic value of SIRI in patients with advanced LUAD patients treated with first‐line chemo‐ or targeted therapy has rarely been reported, and the role of inflammatory markers including SIRI and LDH in predicting efficacy has not been reported.

## MATERIALS AND METHODS

### Patients

We retrospectively reviewed data from 307 patients with advanced LUAD who were first diagnosed in the Department of Thoracic Oncology of Tianjin Medical University Cancer Institute and Hospital from January 2011 to December 2019 and received regular treatment and completed follow‐up data until disease progression. All tumor staging was assessed according to the American Joint Committee on Cancer guidelines, 8th edition.^20^ The inclusion criteria were (1) pathologically diagnosed primary LUAD, (2) stage IV or unresectable stage III according to the TNM staging system, (3) the physical status score is 0–2 according to the Eastern Cooperative Oncology Group (ECOG), (4) complete clinical, laboratory, imaging, and follow‐up data, (5) had not received any antitumor therapy prior to diagnosis, (6) no anti‐inflammatory drugs or immunosuppressants were used, (7) treated with first‐line chemotherapy or targeted therapy regularly until the disease progressed. The exclusion criteria were (1) had a second primary malignancy or chronic inflammatory diseases, (2) clinical evidence of active infection or inflammation, (3) hematological disease or autoimmune diseases, and (4) insufficient clinical or laboratory data. Meanwhile, blood samples from 410 healthy controls and 415 patients with benign pulmonary diseases (inflammatory pseudotumor, tuberculoma, granulation tumor, etc.) and without a history of cancer or active infection or inflammation were used as the control groups.

### Data collection

The clinicopathological characteristics of the patients were collected from the medical records, including sex, age, smoking history, past medical history, family history, TNM stage, gene mutation status, and treatment. Hematological parameters of the patients were collected within 1 week before treatment, at the time of optimal response and disease progression, including white blood cells (WBC), neutrophils (NEUT), lymphocytes (Lym), monocytes (Mon), and platelet (PLT) count as well as LDH and albumin (Alb). The same peripheral blood data were collected from 410 healthy subjects and 415 patients with benign lung disease as controls. This study was approved by the ethics committee of Tianjin Medical University Cancer Institute and Hospital. Patient consent was waived due to the retrospective nature of this paper. This study was conducted according to the Declaration of Helsinki.

The NLR, MLR, PLR and SIRI were calculated as follows: NLR = neutrophils count/lymphocytes count, MLR = monocytes count/lymphocytes count, PLR = platelets count/lymphocytes count, and SIRI = neutrophils count × monocytes count/lymphocytes count.

### Follow‐up

Follow‐up was performed by periodical laboratory analysis every one cycle (3 weeks) and computed tomography or magnetic resonance imaging scans every two cycles (6 weeks). Treatment efficacy was evaluated according to RECISTl.1 standard,^21^ which was divided into complete remission, partial remission, stable disease, and progression disease. The time of optimal efficacy was defined as when the target lesion was minimized during first‐line treatment (the optimal outcome data for this patient were censored if the disease progresses at the first follow‐up after treatment; if successive imaging scans presented stable disease, the time point with the lowest level of tumor markers was defined as the time for obtaining the best therapeutic effect). Progression free survival (PFS) was defined as the time from the start of first‐line therapy to progression or death.

### Statistical analysis

All statistical analyses were performed using SPSS 25.0 statistical software, GraphPad Prism 8.0, and R statistical software. Data were expressed as mean ± standard deviation or median and inter‐quartile range (IQR). One‐way analysis of variance and the Kruskal–Wallis H test were used for comparison between groups. The optional cut‐off values of the laboratory test indicators for distinguishing prognosis were determined by calculating the area under the receiver operating characteristic (ROC) curve (AUC), using the maximum principle of the Youden index. The “Matchit” package in R studio was used to conduct propensity score matching (PSM) analysis, with a matching ratio of 1:1 and a caliper value of 0.01. Survival states were analyzed by the Kaplan–Meier method and compared by log‐rank test. Univariate and multivariate Cox regression proportional hazards models were performed to determine the risk factors affecting the prognosis of LUAD patients, with hazard ratio (HRs) and 95% confidence intervals (95% CIs) expressing the intensity of correlations between the observed factors and PFS. A two‐sided *p* < 0.05 was considered statistically significant.

## RESULTS

### Comparison of inflammation index levels of the three groups and LUAD patients in different treatment periods

A total of 307 LUAD patients were enrolled in this study, whose median age was 58, including 185 (60.3%) males and 122 (39.7%) females. Peripheral blood results of 410 healthy subjects (112 males, 298 females, median age 54 years) and 415 benign lung disease patients (216 males, 199 females, median age 54 years) were collected as controls. By comparing the inflammatory data of the three groups, we found that the pretreatment peripheral blood cells (WBC, NEUT, Mon, and PLT) and the complex indexes based on their count, namely, NLR, MLR, PLR, and SIRI, were higher in LUAD patients. The Lym was lower in LUAD patients than in controls, and the difference was statistically significant (*p* < 0.001). There was no significant difference in these indexes between healthy subjects and patients with benign lung diseases. The level of Alb was the highest in healthy subjects, decreased in patients with benign lung disease, and was the lowest in patients with LUAD, and the differences were statistically significant (*p* < 0.001). The LDH level was lowest in healthy population, increased in benign lung disease, and reached a peak in LUAD patients (*p* < 0.001) (Table 1).

**TABLE 1:** Comparison of inflammatory indexes in healthy subjects, patients with benign lung diseases, and patients with LUAD (mean ± standard deviation)

Group	Number	Sex	Median age (range)	WBC	NEUT	Lym	Mon	PLT	NLR	MLR	PLR	SIRI	Alb	LDH
		No. of males (%)												
		No. of females (%)												
Healthy controls	410	112 (27.3)	54 (39–75)	6.05 ± 1.35	3.52 ± 1.36	2.04 ± 0.68	0.44 ± 0.13	242.24 ± 60.10	1.89 ± 0.90	0.23 ± 0.09	129.09 ± 47.33	0.83 ± 0.45	46.33 ± 1.86	172.03 ± 32.29
		298 (72.7)												
Benign lung diseases	415	216 (52.0)	54 (26–76)	6.00 ± 1.49	3.41 ± 1.15	1.97 ± 0.60	0.44 ± 0.14	236.25 ± 60.84	1.90 ± 1.35	0.24 ± 0.11	129.44 ± 49.23	0.87 ± 0.89	43.56 ± 3.04	184.29 ± 32.64
		199 (48.0)												
LUAD	307	185 (60.3)	58 (29–77)	7.56 ± 2.44	5.09 ± 2.07	1.71 ± 0.55	0.52 ± 0.22	296.94 ± 88.00	3.30 ± 1.85	0.32 ± 0.14	191.92 ± 87.26	1.74 ± 1.42	40.98 ± 4.53	230.83 ± 90.43
		122 (39.7)												
*p* value			*p* < 0.001, *p*1 = 0.535, *p*2 < 0.001, *p*3 < 0.001	*p* < 0.001, *p*1 = 0.934, *p*2 < 0.001, *p*3 < 0.001	*p* < 0.001, *p*1 = 0.457, *p*2 < 0.001, *p*3 < 0.001	*p* < 0.001, *p*1 = 0.339, *p*2 < 0.001, *p*3 < 0.001	*p* < 0.001, *p*1 = 0.967, *p*2 < 0.001, *p*3 < 0.001	*p* < 0.001, *p*1 = 0.397, *p*2 < 0.001, *p*3 < 0.001	*p* < 0.001; *p*1 = 0.996; *p*2 < 0.001; *p*3 < 0.001	*p* < 0.001; *p*1 = 0.656; *p*2 < 0.001; *p*3 < 0.001	*p* < 0.001; *p*1 = 0.999; *p*2 < 0.001; *p*3 < 0.001	*p* < 0.001; *p*1 = 0.828; *p*2 < 0.001; *p*3 < 0.001	*p* < 0.001; *p*1 = *p*2 = *p*3 < 0.001	*p* < 0.001; *p*1 = *P*2 = *p*3 < 0.001

*Note*: *p* < 0.05 is considered significant. *p*1 = healthy subjects vs. patients with benign lung diseases; *p*2 = healthy subjects vs patients with LUAD; *p*3 = patients with benign lung diseases vs. patients with LUAD.

*Abbreviations*: Alb, albumin; LDH, lactic dehydrogenase; LUAD, lung adenocarcinoma; Lym, lymphocyte; MLR, monocyte to lymphocyte ratio; Mon, monocyte; NEUT, neutrophil; NLR, neutrophil to lymphocyte ratio; PLR, platelet to lymphocyte ratio; PLT, platelet; SIRI, systemic inflammation response index; WBC, white blood cell.

**FIGURE 1:**
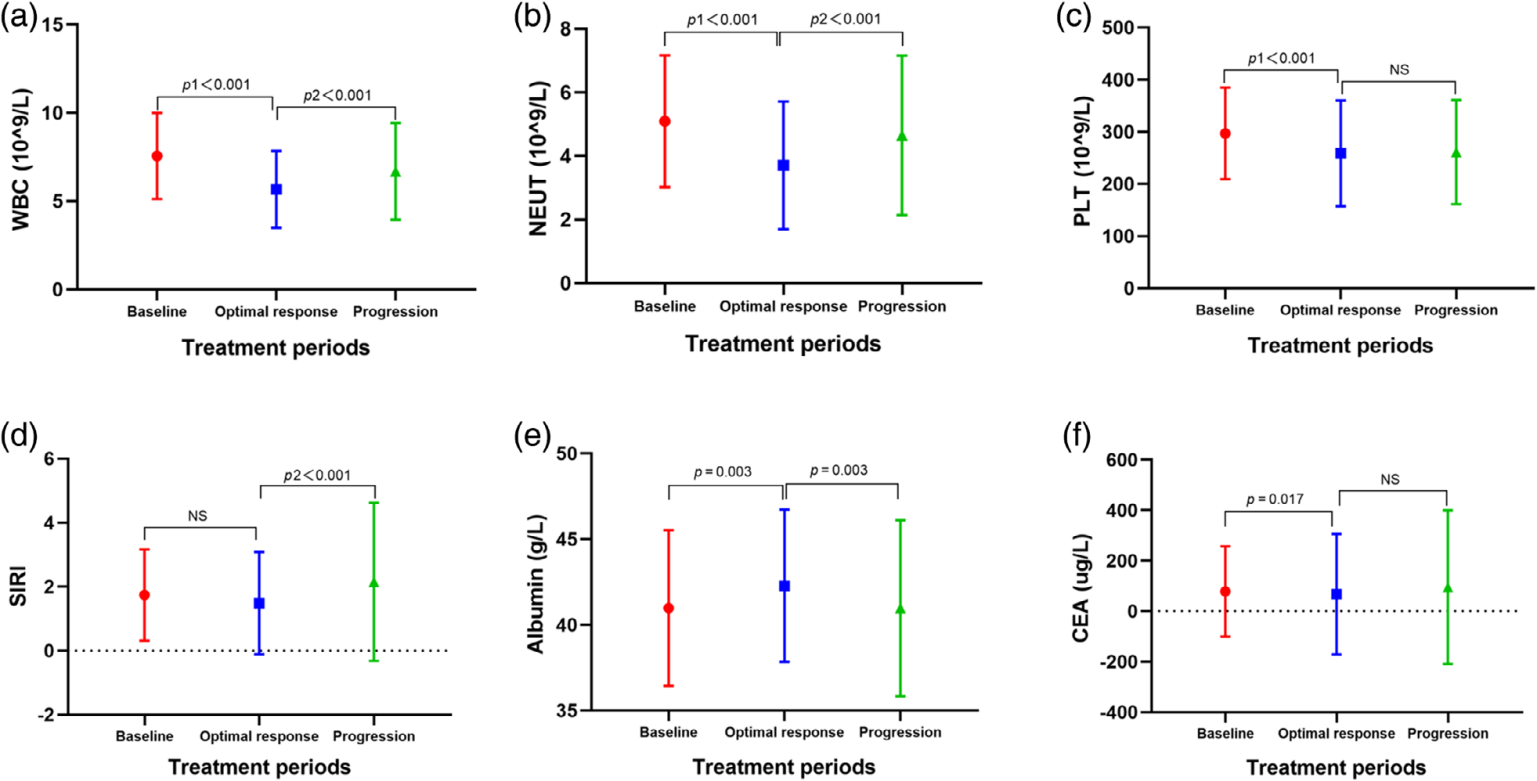
Changes in inflammatory indexes for (a) white blood cell, (b) neutrophil, (c) platelet, (d) systemic inflammation response index, and (e) albumin as well as (f) carcinoembryonic antigen in lung adenocarcinoma patient treatment.

All 307 patients had disease progression during the follow‐up period. Peripheral blood assayed results were collected at optimal response and at disease progression, and inflammatory markers were compared from baseline to optimal response and from optimal response to disease progression. WBC (*p* < 0.001), NEUT (*p* < 0.001), PLT (*p* < 0.001), and SIRI (*p* = 0.001) levels decreased in LUAD patients with optimal response after first‐line therapy and increased again as the disease progressed. However, Alb showed the opposite tendency, which increased when patients obtained the optimal response and decreased as the disease progressed (*p* = 0.019) (see Table 2 and Figure 1).

**TABLE 2:** Comparison of inflammatory indexes (mean ± standard deviation) and CEA (median and IQR) in LUAD patients at baseline, at optimal response, and at disease progression

Treatment response	WBC*	NEUT*	Lym	Mon	PLT*	NLR	MLR	PLR	SIRI*	Alb*	LDH	CEA*
	Mean ± SD	Mean ± SD	Mean ± SD	Mean ± SD	Mean ± SD	Mean ± SD	Mean ± SD	Mean ± SD	Mean ± SD	Mean ± SD	Mean ± SD	Median (IQR)
Baseline	7.56 ± 2.44	5.09 ± 2.07	1.71 ± 0.55	0.51 ± 0.22	296.94 ± 88.00	3.30 ± 1.85	0.32 ± 0.14	191.92 ± 87.26	1.74 ± 1.42	40.98 ± 4.53	230.83 ± 90.43	17.57 (60.680)
Optimal response	5.66 ± 2.18	3.71 ± 2.01	1.38 ± 0.56	0.48 ± 0.26	258.88 ± 101.52	3.45 ± 4.68	0.38 ± 0.25	228.77 ± 244.66	1.49 ± 1.59	42.28 ± 4.44	243.48 ± 68.30	10.94 (26.857)
Progression	6.6 ± 2.74	4.65 ± 2.51	1.38 ± 0.56	0.53 ± 0.28	261.24 ± 99.93	4.03 ± 3.06	0.42 ± 0.27	223.69 ± 138.06	2.15 ± 2.27	40.98 ± 5.13	267.40 ± 107.69	14.10 (52.710)
*p* value	*p* < 0.001; *p*1 < 0.001; *p*2 < 0.001	*p* < 0.001; *p*1 < 0.001; *p*2 < 0.001	*p* < 0.001, *p*1 < 0.001, *p*2 = 1.000	*p* = 0.106	*p* < 0.001, *p*1 < 0.001, *p*2 = 1.000	*p* = 0.002, *p*1 = 0.941, *p*2 = 0.222	*p* < 0.001, *p*1 = 0.002, *p*2 = 0.147	*p* = 0.001, *p*1 = 0.052, *p*2 = 0.986	*p* = 0.001, *p*1 = 0.119, *p*2 < 0.001	*p* = 0.001; *p*1 = 0.003; *p*2 = 0.003	*p* < 0.001; *p*1 = 0.202; *p*2 = 0.011	*p* = 0.019; *p*1 = 0.017; *p*2 = 0.154

*Note*: *p* < 0.05 is considered significant. *p*1 = baseline vs. optimal response; *p*2 = optimal response vs. disease progression.

*Abbreviations*: Alb, albumin; IQR, inter‐quartile range; LDH, lactic dehydrogenase; LUAD, lung adenocarcinoma; Lym, lymphocyte; MLR, monocyte to lymphocyte ratio; Mon, monocyte; NEUT, neutrophil; NLR, neutrophil to lymphocyte ratio; PLR, platelet to lymphocyte ratio; PLT, platelet; SIRI, systemic inflammation response index; WBC, white blood cell.

### Associations between SIRI and clinicopathological characteristics of patients before and after PSM analysis

The collected pretreatment NLR, MLR, PLR, SIRI, and LDH were drawn using ROC curves according to the progression status of patients, and the optimal cut‐offs were determined to be 2.73, 0.32, 141.84, 1.21, and 234 by Youden index. The AUCs were 0.599 (95% CI 0.534–0.664, *p* = 0.004), 0.618 (95% CI 0.555–0.682, *p* = 0.001), 0.566 (95% CI 0.500–0.632, *p* = 0.053), 0.625 (95% CI 0.562–0.689, *p* < 0.001), and 0.596 (95% CI 0.525–0.666, *p* = 0.008), respectively, as shown in Table 3.

**TABLE 3:** Evaluation of the diagnostic value of inflammatory related indicators

Variable	Cut‐off point	Sensitivity	Specificity	*p* value	AUC (95% CI)
NLR	2.73	0.620	0.600	0.004	0.599 (0.534,0.664)
MLR	0.32	0.505	0.730	0.001	0.618 (0.555,0.682)
PLR	141.84	0.745	0.383	0.053	0.566 (0.500,0.632)
SIRI	1.21	0.630	0.583	<0.001	0.625 (0.562,0.689)
LDH	234	0.431	0.760	0.008	0.596 (0.525,0.666)

*Note*: The curve coordinate data table was obtained according to the ROC curve, and the Youden index (sensitivity + specificity − 1) was calculated. The maximum value of the Youden index was the optimal cut‐off.

*Abbreviations*: LDH, lactic dehydrogenase; MLR, monocyte to lymphocyte ratio; NLR, neutrophil to lymphocyte ratio; PLR, platelet to lymphocyte ratio; SIRI, systemic inflammation response index.

Considering SIRI integrates neutrophils, monocytes, and lymphocytes, LDH and some routine clinicopathological features were selected to be included in the subsequent correlation analysis. Table 4 summarizes the correlations between SIRI and clinicopathological characteristics in patients of the primary cohort. In the unmatched complete data set, there were 137 patients with SIRI ≤ 1.21 and 170 patients with SIRI > 1.21. Higher SIRI levels were significantly associated with male gender (*p* = 0.007), smokers (*p* = 0.001), patients who didn't respond to treatment (*p* = 0.048), and elevated PLR (*p* = 0.001) and LDH (*p* < 0.001). In the 1:1 matched data set (56 patients with SIRI ≤ 1.21, 56 patients with SIRI > 1.21), there were no significant correlations between SIRI and the factors mentioned above.

**TABLE 4:** Baseline clinicopathological characteristics for patients with SIRI ≤ 1.21 vs. SIRI > 1.21 before and after PSM analysis

Characteristic	Pre‐PSM			Post‐PSM		
	SIRI≤1.21 (*n* = 137)	SIRI > 1.21 (*n* = 170)	*p* value	SIRI≤1.21 (*n* = 56)	SIRI > 1.21 (*n* = 56)	*p* value
Sex			0.007			0.571
Male	71	114		31	27	
Female	66	56		25	29	
Age (years)			0.216			0.186
≤58	75	81		25	33	
>58	62	89		31	23	
Smoking history			0.001			1.000
No	79	66		33	34	
Yes	58	104		23	22	
Family history			0.112			1.000
No	101	111		44	43	
Yes	36	59		12	13	
TNM stage			0.301			1.000
III	18	16		5	6	
IV	119	154		51	50	
Treatment			0.784			0.625
Chemo‐based on PEM	104	131		44	41	
Chemo‐based on PTX	18	25		7	9	
Chemo‐combined with TKIs	13	11		4	6	
Others	2	3		1	0	
Optimal treatment response			0.048			0.802
SD	98	101		38	35	
PR	32	50		16	18	
PD	7	19		2	3	
PLR (mean)			0.001			0.834
≤141.84	55	38		17	15	
>141.84	82	132		39	41	
LDH (mean)			<0.001			1.000
≤234	96	79		40	40	
>234	26	73		16	16	

*Note*: *p* < 0.05 is considered significant.

*Abbreviations*: LDH, lactic dehydrogenase; PD, progression disease; PEM, pemetrexed disodium; PLR, platelet to lymphocyte ratio; PR, partial remission; PSM: propensity score matching; PTX, paclitaxel liposome; SD, stable disease; SIRI, systemic inflammation response index; TKIs, tyrosine kinase inhibitors.

### Correlations between NLR, MLR, PLR, SIRI, LDH, and patient survival

**FIGURE 2:**
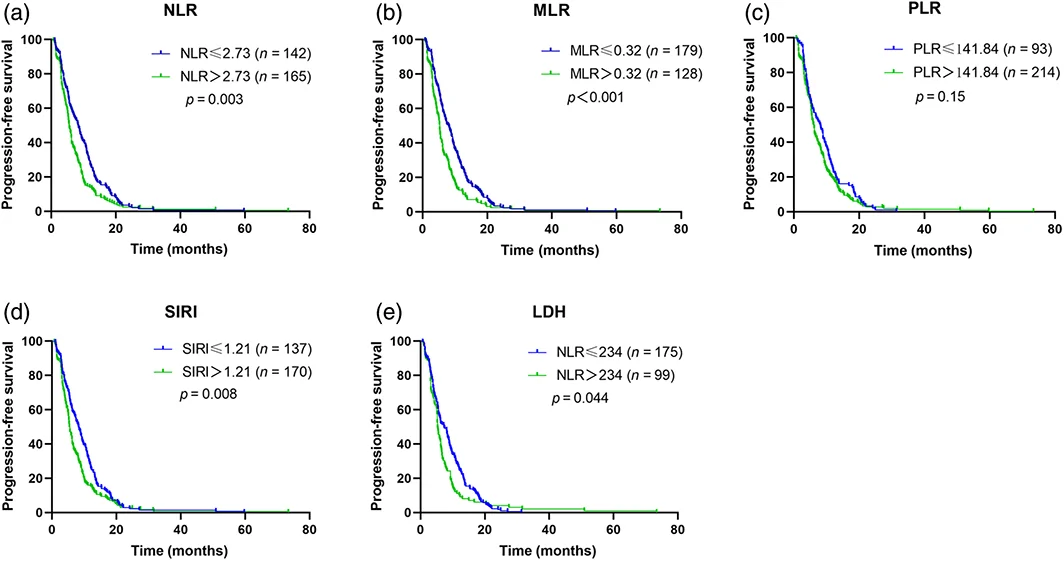
Kaplan–Meier curves for PFS according to (a) the neutrophil to lymphocyte ratio (NLR), (b) the monocyte to lymphocyte ratio (MLR), (c) the platelet to lymphocyte ratio (PLR), (d) the systemic inflammation response index (SIRI), and (e) lactic dehydrogenase (LDH). A high (>2.73) NLR and high (>0.32) MLR as well as high (>1.21) SIRI and high (>234) LDH are associated with significantly shorter PFS.

To evaluate the relationships between inflammatory indicators and patient survival, the survival curves of PFS in the complete data set were estimated using the Kaplan–Meier method and compared by log‐rank test. The results are shown in Figure 2a–e. High levels of NLR, MLR, SIRI, and LDH are all associated with poor PFS (all *p <* 0.05). An elevated PLR level was also associated with poor PFS, but the difference was not statistically significant (*p* = 0.15).

### Univariate and multivariate Cox analysis of PFS

As see from Table 5, univariate survival analysis showed that in the unmatched complete data set, treatment (*p* < 0.001), optimal treatment response (*p* < 0.001), SIRI (*p* = 0.009), and LDH (*p* = 0.045) could significantly affect the PFS of LUAD patients. However, the level of PLR didn't affect patients' PFS. Other factors, such as sex, age, smoking history, family history, and stage were not identified to influence PFS (all *p* > 0.05). Multivariate analysis was conducted on the abovementioned inflammation‐based prognostic index and indicated that treatment (*p* = 0.023), optimal treatment response (*p* < 0.001), SIRI (*p* = 0.028), and LDH (*p* = 0.003) were still independent prognostic factors for PFS.

**TABLE 5:** Univariate and multivariate COX proportional hazards analyses of PFS in LUAD patients before PSM analysis

Clinical characteristic	Number	Median PFS (95% CI)	Univariate analysis		Multivariate analysis	
			HR (95% CI)	*p* value	HR (95% CI)	*p* value
Sex				0.511		0.405
Male	185	6.30 (5.54–7.05)	1 (reference)			
Female	122	6.30 (4.83–7.77)	0.926 (0.736–1.165)			
Age (years)				0.144		0.625
≤ 58	156	7.07 (5.66–8.47)	1 (reference)			
>58	151	5.63 (4.94–6.32)	1.183 (0.944–1.482)			
Smoking history				0.330		0.115
No	145	7.50 (6.03–8.97)	1 (reference)			
Yes	162	5.83 (5.15–6.51)	1.118 (0.893–1.401)			
Family history				0.546		0.721
No	212	6.33 (5.14–7.52)	1 (reference)			
Yes	95	6.00 (5.28–6.72)	0.928 (0.726–1.185)			
TNM stage				0.864		0.728
III	34	6.23 (4.14–8.33)	1 (reference)			
IV	273	6.30 (5.42–7.17)	0.969 (0.678–1.386)			
Treatment						
Chemo‐based on PEM	235	6.30 (5.66–6.94)	1 (reference)		1 (reference)	
Chemo‐based on PTX	43	5.00 (2.99–7.01)	1.264 (0.913–1.751)	0.267	1.187 (0.831–1.696)	0.055
Chemo‐combined with TKIs	24	12.97 (9.77–16.17)	0.430 (0.275–0.672)	0.003	0.402 (0.241–0.671)	0.070
Others	5	3.60 (2.96–4.24)	2.279 (0.935–5.552)	0.075	1.132 (0.444–2.881)	0.207
Optimal treatment response						
SD	199	6.33 (5.26–7.41)	1 (reference)		1 (reference)	
PR	82	7.90 (6.51–9.29)	0.815 (0.629–1.057)	0.123	0.799 (0.595–1.073)	0.149
PD	26	1.40 (1.29–1.51)	9.751 (6.330–15.022)	<0.001	18.668 (10.759–32.389)	<0.001
PLR (mean)			1.197 (0.936,1.529)	0.151		0.810
≤ 141.84	93	8.13 (6.10, 10.17)				
>141.84	214	6.00 (5.48, 6.52)				
SIRI (mean)			1.354 (1.079–1.699)	0.009	1.565 (1.188–1.062)	0.028
≤1.21	137	8.30 (6.90–9.70)				
>1.21	170	5.43 (4.87–5.99)				
LDH (mean)			1.295 (1.005–1.668)	0.045	1.409 (1.067–1.861)	0.003
≤234	175	7.30 (5.77–8.83)				
>234	99	5.30 (4.70–5.90)				

*Note*: *p* < 0.05 is considered significant.

*Abbreviations*: LDH, lactic dehydrogenase; PD, progression disease; PEM, pemetrexed disodium; PLR, platelet to lymphocyte ratio; PR, partial remission; PSM: propensity score matching; PTX, paclitaxel liposome; SD, stable disease; SIRI, systemic inflammation response index; TKIs, tyrosine kinase inhibitors.

In the 1:1 matched data set, univariate survival analysis showed that only optimal treatment response (*p* < 0.001) and LDH (*p* = 0.07) affected prognosis. Further multivariate analysis indicated that treatment (*p* = 0.007), optimal treatment response (*p* < 0.001), SIRI (*p* = 0.013), and LDH (*p* = 0.034) were significantly associated with PFS (Table 6), which accorded with the conclusion before PSM.

**TABLE 6:** Univariate and multivariate COX proportional hazards analyses of PFS in LUAD patients after PSM analysis

Clinical characteristic	Number	Median PFS (95% CI)	Univariate analysis		Multivariate analysis	
			HR (95% CI)	*p* value	HR (95% CI)	*p* value
Sex				0.261		0.159
Male	58	8.90 (7.57–11.69)	1 (reference)		1 (reference)	
Female	54	7.50 (6.64–9.56)	1.24 (0.85–1.80)		1.50 (0.85–2.62)	
Age (years)				0.730		0.380
≤58	58	8.10 (7.22–11.32)	1 (reference)		1 (reference)	
>58	54	8.35 (6.98–9.99)	1.07 (0.73–1.56)		1.20 (0.80–1.82)	
Smoking history				0.773		0.473
No	67	8.20 (7.30–9.96)	1 (reference)		1 (reference)	
Yes	45	8.30 (6.75–11.81)	0.95 (0.65–1.39)		1.22 (0.71–2.07)	
Family history				0.425		0.906
No	87	8.00 (7.17–10.13)	1 (reference)		1 (reference)	
Yes	25	8.70 (7.17–12.29)	0.83 (0.53–1.30)		0.97 (0.54–1.72)	
TNM stage				0.600		0.265
III	11	8.30 (4.95–14.41)	1 (reference)		1 (reference)	
IV	101	8.20 (7.47–10.14)	1.18 (0.63–2.21)		1.55 (0.72–3.36)	
Treatment						
Chemo‐based on PEM	85	8.20 (7.24–10.18)	1(reference)		1 (reference)	
Chemo‐based on PTX	16	6.20 (4.58–11.65)	1.05 (0.61–1.81)	0.857	1.20 (0.67–2.15)	0.546
Chemo‐combined with TKIs	10	12.30 (7.47–16.95)	0.60 (0.31–1.17)	0.143	0.34 (0.15–0.74)	0.007
Others	1	3.3	4.18 (0.56–31.06)	0.160	8.32 (1.01–68.69)	0.049
Optimal treatment response						
SD	73	8.30 (7.40–10.72)	1(reference)		1 (reference)	
PR	34	8.55 (7.61–11.72)	0.91 (0.60–1.36)	0.632	0.95 (0.62–1.48)	0.830
PD	5	1.20 (0.92–1.52)	227.25 (25.3–2041.5)	<0.001	143.07 (15.08–1357.50)	<0.001
PLR (mean)				0.984		
≤141.84	32	9.00 (6.94–10.74)	1 (reference)		1 (reference)	0.411
>141.84	80	7.75 (7.28–10.54)	1.01 (0.67–1.52)		0.81 (0.48–1.35)	
SIRI (mean)				0.160		
≤1.21	56	9.20 (7.83–11.94)	1 (reference)		1 (reference)	0.013
>1.21	56	6.30 (6.39–9.40)	1.31 (0.90–1.91)		1.69 (1.11–2.58)	
LDH (mean)				0.073		0.034
≤234	80	9.00 (8.19–10.66)	1 (reference)		1 (reference)	
>234	32	5.30 (4.26–10.86)	1.48 (0.97–2.25)		1.75 (1.04–2.93)	

*Abbreviations*: LDH, lactic dehydrogenase; PD, progression disease; PEM, pemetrexed disodium; PLR, platelet to lymphocyte ratio; PR, partial remission; PSM: propensity score matching; PTX, paclitaxel liposome; SD, stable disease; SIRI, systemic inflammation response index; TKIs, tyrosine kinase inhibitors.

### Prognostic value of SIRI combined with LDH

**FIGURE 3:**
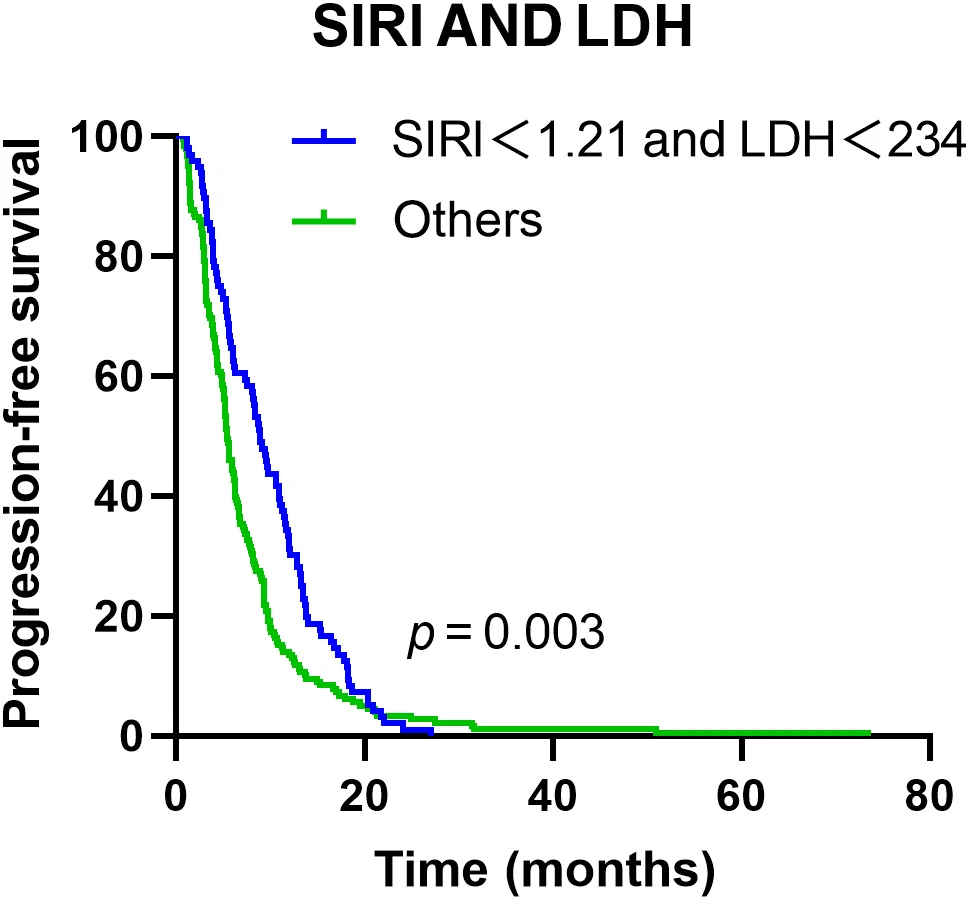
Kaplan–Meier curves for PFS according to different levels of systemic inflammation response index (SIRI) combined with lactic dehydrogenase (LDH). Low (≤1.21) SIRI and low (≤234) LDH are associated with better PFS.

**FIGURE 4:**
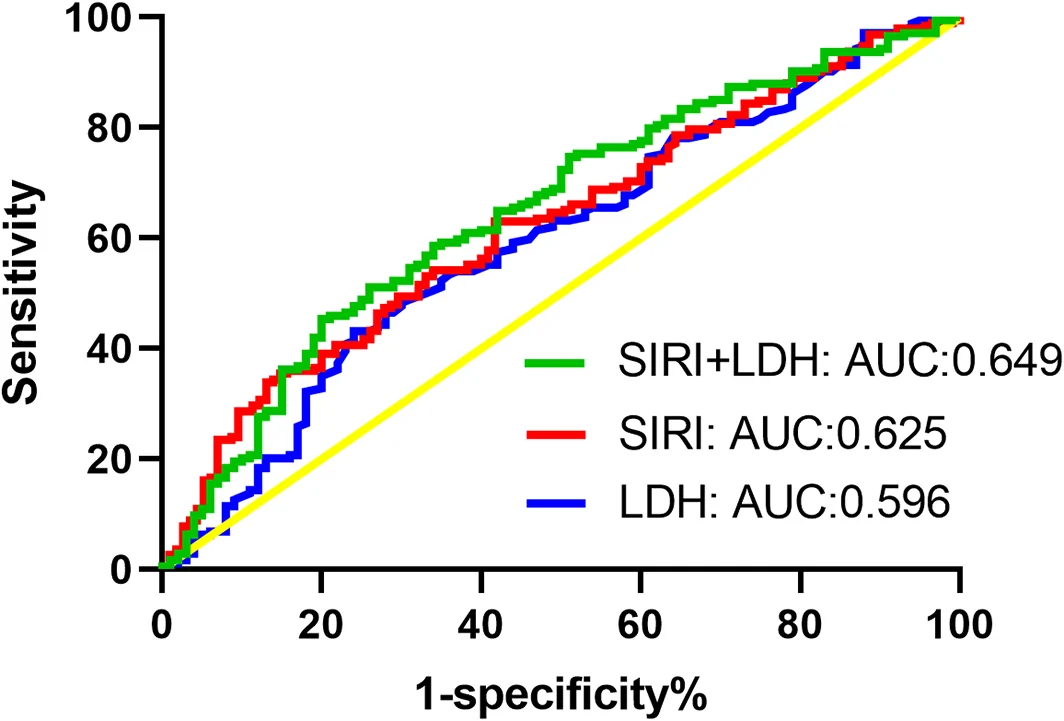
Receiver operating characteristic curve analysis of systemic inflammation response index (SIRI) and lactic dehydrogenase (LDH) alone or a combination of SIRI and LDH. In advanced LUAD patients, the area under the curves (AUCs) of SIRI and LDH were 0.625 and 0.596. When SIRI and LDH were combined, AUC increased to 0.649.

Multivariate Cox analysis showed that SIRI > 1.21 and LDH > 234 were independent prognostic factors for PFS. Patients were further divided into two groups based on these two risk factors: SIRI ≤ 1.21 and LDH ≤ 234 (low‐risk group); SIRI > 1.21 and LDH ≤ 234, SIRI ≤ 1.21 and LDH > 234, SIRI > 1.21 and LDH > 234 (high‐risk group). Kaplan–Meier curves showed that the median progression free survival of patients in the low‐risk group was significantly longer than that of patients in the high‐risk group (8.87 vs. 5.43 months) (Figure 3). SIRI, LDH, and the combination of these two indexes were enrolled into ROC analysis, where the AUCs reached 0.625 (95% CI 0.562–0.689, *p <* 0.001), 0.596 (95% CI 0.525–0.666, *p* = 0.008), and 0.649 (95% CI: 0.582–0.717, *p* < 0.001), respectively (Figure 4).

## DISCUSSION

In this study, the clinical data of 307 advanced LUAD patients who were treated in our department and received regular treatment in recent 10 years were retrospectively analyzed. The data of 410 healthy individuals and 415 patients with benign lung diseases were collected as controls. The study showed that pretreatment SIRI and other inflammatory indicators in patients with advanced LUAD were higher than those in healthy population and patients with benign lung disease. The dynamic changes in SIRI during treatment could reflect therapeutic efficacy. SIRI may serve as a novel and independent factor for predicting PFS, outperforming NLR and MLR. Additionally, the combination of SIRI and LDH may be a better prognostic stratification factor for LUAD patients.

It is widely recognized that inflammation plays a fundamental role in pathogenesis and tumor progression in patients with many solid tumors, which possibly relates to changes in the tumor immune microenvironment.^22^ Moreover, tumor immune microenvironment changes are closely associated with inflammatory and immune cell distribution in the peripheral blood.^23^, ^24^ Some studies have suggested that abnormal changes in systemic inflammatory cells, such as neutrophils, monocytes, and lymphocytes, are linked to the prognosis of many malignancies. Neutrophils could influence tumor development and progression via promoting tumor angiogenesis, aiding tumor cells to evade immune surveillance, and secreting large amounts of reactive oxygen species and nitric oxide.^25^, ^26^, ^27^ Monocytes were found to differentiate into tumor‐associated macrophages, which could secrete tumor necrosis factor alpha and vascular endothelial growth factor to facilitate tumor angiogenesis, inflammatory response, and metastases.^28^, ^29^ Lymphocytes are crucial components of anticancer immunity and immune surveillance.^30^ Downregulation of peripheral lymphocytes impairs the host's anticancer immunity and correlates with poor survival in cancer patients.^31^ SIRI is a more potent prognosticator due to fully assessing the balance between host immune and inflammatory conditions by integrating various cell types simultaneously. A recent meta‐analysis that included 14 studies from 2016 to 2020 involving pancreatic cancer, gastric cancer, liver cancer, esophageal cancer, nasopharyngeal cancer, renal cancer, NSCLC, upper tract urothelial carcinoma, and breast cancer showed that high SIRI levels were associated with poor overall survival (HR 2.20, 95% CI 1.85–2.62, *p*  < 0.001) and PFS (HR 1.73, 95% CI 1.38–2.16, *p*  <  0.001).^32^

However, the prognostic value of SIRI in advanced LUAD patients has rarely been reported, and the predictive effect of SIRI and other inflammatory markers on treatment efficacy has not been reported. Our results showed that pretreatment inflammatory markers such as NLR, MLR, PLR, and SIRI in LUAD patients were higher than those in healthy subjects and patients with benign lung diseases, and the lymphocyte count was lower. However, there was no obvious difference between healthy patients and benign lung disease patients, which to some extent confirmed the role of inflammatory indicators in the progression of LUAD. Zhu et al.^33^ also reported that NLR and PLR could be used as diagnostic markers for lung cancer patients to distinguish healthy subjects. In addition, we found that serum albumin and LDH were significantly different among the three groups, suggesting the role of albumin and LDH in the occurrence and development of pulmonary disease, especially LUAD. LDH facilitates the glycolytic process by converting pyruvate to lactate, which is produced by rapidly growing tumors and therefore reflects the tumor burden.^34^ Additionally, Fiala O et al.^35^ suggested that reduced serum albumin was associated with poor outcomes for NSCLC. To our knowledge, few studies have reported whether inflammatory factors such as SIRI as well as albumin and LDH could be used as diagnostic biomarkers for advanced LUAD. Therefore, this study revealed that pretreatment hematological parameters could help to distinguish LUAD patients from nontumor patients.

In addition, some inflammatory markers (WBC, NEUT, PLT, and SIRI) decreased in LUAD patients with optimal response after first‐line therapy and increased again as the disease progressed. These findings suggest that inflammatory indexes are related to the dynamic development of the disease. The dynamic monitoring of these inflammatory markers might help to predict the short‐term treatment effect, detect the tendency of drug resistance as early as possible, and enable timely adjustment of the treatment strategy. Additionally, we found that changes in albumin during treatment showed the opposite tendency, suggesting that the nutritional status of patients could reflect changes in the disease. Serum albumin has been described as an independent prognosticator of survival in various cancers. As early as 2010, a systematic review revealed that high albumin levels are associated with better outcomes in different types of cancer.^36^ Changes in CEA, currently the most widely used tumor biomarker of LUAD, further verified the above conclusion. A meta‐analysis indicated that changes in CEA levels during treatment in patients with NSCLC could predict outcomes.^37^ There have been few studies that have dynamically monitored the inflammation index to predict the therapeutic response. In this study, we examined the predictive effect of inflammatory indicators, including SIRI and albumin, on the treatment efficacy of LUAD patients. These noninvasive and easily accessible indexes have the potential to be effective biomarkers in efficacy prediction and further help clinicians decide on the optimal treatment strategy.

We also evaluated the prognostic value of pretreatment inflammatory markers (NLR, MLR, PLR, and SIRI) and LDH in patients with advanced LUAD. The results showed that higher baseline levels of NLR, MLR, SIRI, and LDH predicated worse PFS. The Cox proportional hazards model confirmed that SIRI and LDH are correlated with PFS in both the data sets before and after PSM analysis. Furthermore, compared with NLR or MLR, the AUC of SIRI was larger. These results demonstrate the prediction ability of SIRI and its potential superiority over NLR and MLR. This is similar to the findings of Hu et al.^38^ that SIRI is an independent prognostic factor in unresectable stage III NSCLC patients who undergo chemoradiotherapy, with a superior prognostic value than NLR. The study by Kucuk et al.^39^ also suggested that SIRI was an independent predictor of OS for limited‐stage SCLC treated with concurrent chemoradiotherapy. Unlike previous studies, our research showed that high pretreatment SIRI level was significantly associated with shorter PFS in advanced LUAD patients, and the effect of other NSCLC histological subtypes on the results was ruled out. In addition to SIRI, the present study also indicated that LDH was a significant prognostic factor for PFS. Several previous studies reported that there was a significant association between high serum LDH level and poor survival in NSCLC patients receiving standard chemotherapy, tyrosine kinase inhibitors (TKIs), or immune checkpoint inhibitors.^40^, ^41^, ^42^, ^43^ We performed further stratified analyses based on these two risk factors. We found that patients with SIRI ≤ 1.21 and LDH ≤ 234 had significantly longer PFS than those in the other subgroups. Further analysis showed in high risk group, there were two patients harboring sensitive epidermal growth factor receptor (EGFR) mutations received combined treatment of EGFR‐TKIs (erotinib) and platinum‐based chemotherapy, with PFSs up to 31.6 and 73.4 months, respectively. Another patient with an EGFR mutation had a PFS of 30 months after receiving six cycles of pemetrexed plus platinum‐based chemotherapy followed by gefitinib maintenance. In addition, an anaplastic lymphoma kinase (ALK)‐positive patient was treated with crizotinib and achieved a PFS of 50.9 months. These results are consistent with previous studies that showed that TKIs significantly improved outcomes in LUAD patients with EGFR and ALK mutations.^44^ The longer PFS of the four patients from the high‐risk group may help to explain why the two groups' survival curves cross. The ROC curves further showed that the combination of SIRI and LDH could help to increase predictive value of LUAD patients' prognosis, presenting its capability to be a clinically accessible biomarker.

The present study has several limitations. First, it was a single‐center retrospective study with a small sample size and potential bias that could affect the results, therefore a large‐scale, multicenter, prospective cohort study is needed to confirm our results. Second, available data on outcomes in the immunotherapy population are limited, which also limits our exploration of the potential value that SIRI and other inflammatory markers have as predictive markers of immunotherapy.

## CONCLUSION

In summary, our study demonstrated that pretreatment peripheral blood SIRI is an independent predictor of PFS in advanced LUAD patients. Dynamic monitoring of inflammatory indexes changes during treatment could help predict therapeutic efficacy. As a novel prognostic marker, the prognostic value of SIRI was superior to that of NLR and MLR. Moreover, we found that the combination of SIRI and LDH could be a better predictive marker for LUAD patient prognosis.

## AUTHOR CONTRIBUTIONS

Peng Chen, Ran Zuo, Fuyi Zhu, and Cuicui Zhang designed the study and performed overall data interpretation. Jincheng Ma and Jinliang Chen were responsible for data collection, assembly, and data analysis. Ping Yue, Jinfang Cui, and Yu Wang were responsible for writing and revising the manuscript. All authors read and critically revised the manuscript for intellectual content and approved the final manuscript.

## CONFLICT OF INTEREST STATEMENT

The authors declare no conflict of interest.

## Data Availability

All data are fully available without restrictions.
